# Making genome browsers portable and personal

**DOI:** 10.1186/s13059-018-1470-9

**Published:** 2018-07-18

**Authors:** Aziz Khan, Xuegong Zhang

**Affiliations:** 1 Centre for Molecular Medicine Norway (NCMM), Nordic EMBL Partnership, University of Oslo, 0318 Oslo, Norway; 2 Key Lab of Bioinformatics/Bioinformatics Division, BNRIST (Beijing National Research Center for Information Science and Technology), Department of Automation, Tsinghua University, Beijing, 100084 China; 3 School of Life Sciences, Tsinghua University, Beijing, 100084 China

**Keywords:** Genome browser, Visualization, Genomic interaction, Algorithm

## Abstract

GIVE is a framework and library for creating portable and personalized genome browsers. It makes visualizing genomic data as easy as building a laboratory homepage.

## Introduction

In the genomic and post-genomic era, biology is fast becoming a data science. The amount of genomic data is increasing dramatically, boosted by the latest advancements in single-cell sequencing, as well as ChIP-seq (chromatin immunoprecipitation sequencing), Hi-C, ATAC-seq and so on. Efficient visualization of these types of data and the networks between them is becoming more demanding and challenging even with the rapid development of new visualization methods. Since the early days of the Human Genome Project, web-based genome browsers have been developed [1], with the Ensembl [2] and UCSC [3] genome browsers being the longest maintained and most widely used. However, such centralized genome browsers cannot meet the ever-increasing needs for customized visualization of diverse types of data and cannot be used for increasingly diverse types of analyses. Many individual researchers, as well as larger laboratories, are eager to visualize and publicize genomic, epigenomic and transcriptomics data in their specific area of investigation, but building genome browsers is not always an easy task. In a recent article [4] published in *Genome Biology*, Cao et al. of Dr. Sheng Zhong’s laboratory at the University of California San Diego (UCSD), USA, described a novel programming library that was named GIVE (genomic interaction visualization engine) for creating portable and versatile genome browsers that can be used on personal websites. GIVE enables non-expert website developers to equip their websites with versatile features to visualize and analyse multiple types of genomic data, such as genome annotation, and linear and quantitative data, as well as interactions between multiple types of data and data from different genomic locations. With the library and tools provided by GIVE, building a laboratory website that contains different pages or windows that show highly personalized and interactive views of genomic data can be as straightforward as, for example, building a hotel webpage with an embedded Google map.

## Easily setting up a genome browser on a personal webpage

**Fig. 1:**
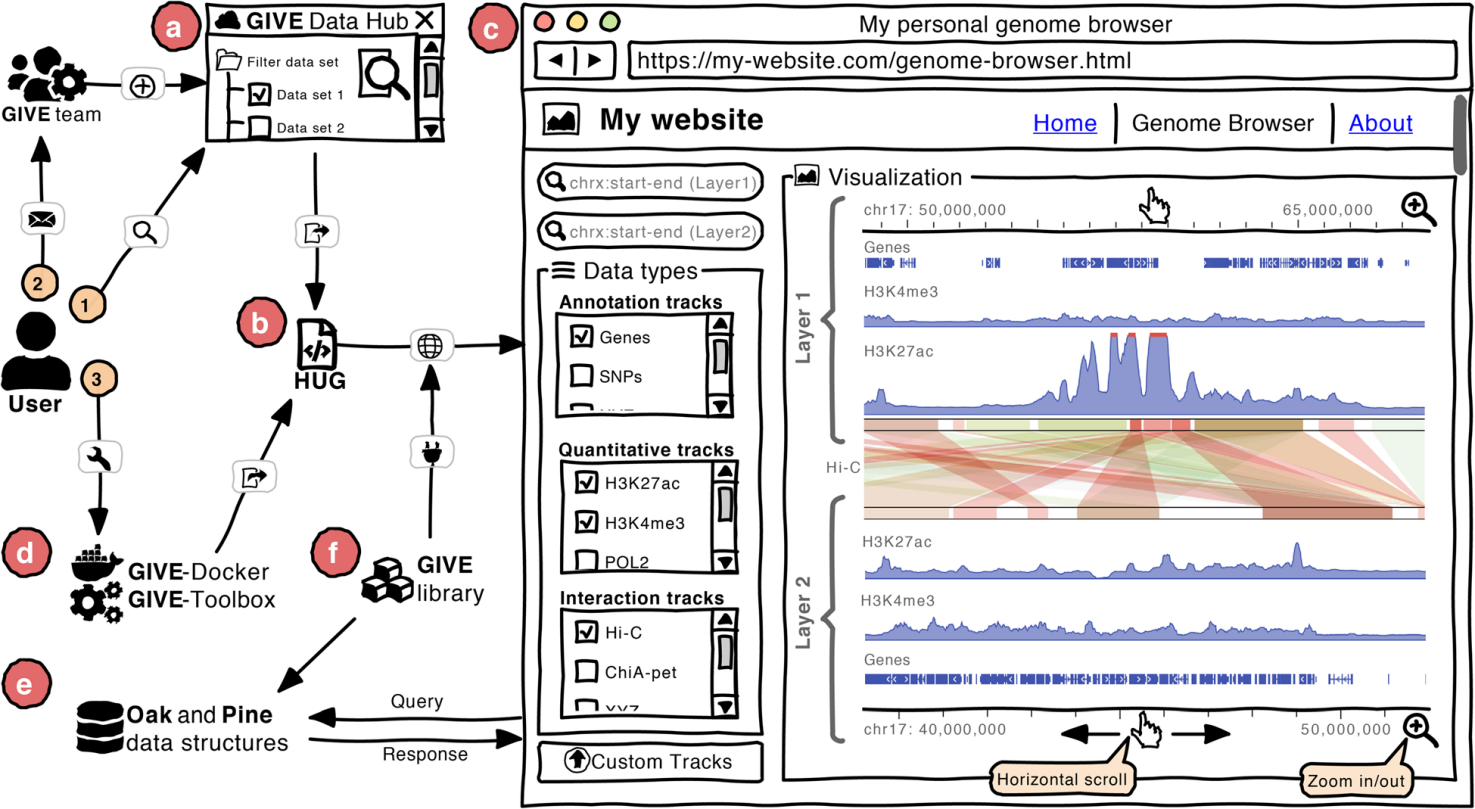
A schematic representation of the different features of GIVE. **a** The GIVE (genomic interaction visualization engine) Data Hub, a central repository maintained by the GIVE team, enables a user to search for data of interest and export it as embeddable HTML. **b** HUG (HTML universal generator) is a graphical interface on the Data Hub that automatically generates HTML code for selected data that can be incorporated into any website. **c** An example of the GIVE genome browser user interface after embedding the code generated by HUG into a website is shown. This example shows several of the available options, including double-layer display, data selection checkboxes, and scrolling and zooming options. **d** GIVE-Toolbox and GIVE-Docker can be used to quickly set up a local version of the GIVE server. **e** Oak and Pine are tree-based data structures that seamlessly transfer data from the GIVE server to the web browser in response to a query requested by the user. **f** The portable GIVE library uses web components for data transfer and visualization and encapsulates data structures algorithms Oak and Pine

Recent advances in web technologies have made it possible to develop interactive, reusable and modular web applications, but the full realization of such possibilities by small laboratories without web development expertise can often be a challenge. In their recent article, Cao et al. described how they succeeded in filling this knowledge gap by developing an open source HTML5 and JavaScript library to create portable genome browsers that can be shared on personal websites. GIVE uses web components — a set of web platform application programming interfaces — to create reusable and encapsulated HTML tags. Figure 1 is a schematic representation of the GIVE library and its features. The GIVE framework provides an easy way to set up a genome browser by adding a few lines of HTML tags and by using data hosted on any publicly available server. The GIVE library supports three types of data tracks: genome annotations in BED format, quantitative data in Wig/BigWig format and genome interactions in interaction matrix format.

Scientists often need to visualize and analyse their own in-house data together with data available in the public domain and/or to collect multiple sources of public data to perform their own analyses. The scientific community actively shares generated data with the public by creating tracks or track-hubs, such as those collated in the Track Hub Registry (https://trackhubregistry.org/). To help facilitate data sharing, the developers of GIVE made several public data sets available through the GIVE Data Hub, which is a central metadata repository with searching, filtering and exporting features (Fig. 1a). The GIVE Data Hub provides an interactive user interface generator that is named GIVE HUG (HTML universal generator), to enable users to easily and automatically generate embeddable HTML code and to launch a fully functional genome browser without the need for advanced bioinformatics or programming skills (Fig. 1b).

## Bringing distant data into the same frame

One of the key challenges for genome browsers is the visualization of diverse types of data generated by different technologies and of the interactions and relationships between different elements from different genomic regions. For example, it is very difficult to view genome annotation data (such as enhancer regions) with enhancer–promoter interaction data derived from a Hi-C experiment, as these interactions can be either inter-chromosome or intra-chromosome. To address this, GIVE has a double-layer display to easily visualize, compare and analyse genome interactions alongside other genome annotation data. Thus, two parallel genome coordinates can be added to the browser: one at the top and the other at the bottom of the visualization panel. In the example shown in Fig. 1c, the middle section displays the genome interactions from Hi-C data in the MCF-7 cell line, which separates layer 1 and layer 2 of UCSC gene annotations and H3K27ac, H3K4me3 ChIP-seq signals from different regions of chromosome 17 in the human genome. The displays of the top and bottom genome coordinates are independent and can be easily scrolled horizontally or zoomed in and out to visually compare inter-chromosomal and intra-chromosomal interactions.

## Setting up a local version of the GIVE server

Users with the computational infrastructure and basic programming skills may want to set up their own local versions of GIVE in order to build and customize the genome browser to meet their specific needs. The creators of GIVE have provided GIVE-Toolbox and GIVE-Docker (Fig. 1d) to enable users to easily set up a local version of the GIVE genome browser. GIVE-Toolbox contains a set of command-line scripts necessary to set up the GIVE server by creating the database and data tracks. The GIVE creators recommend the use of GIVE-Docker, which is a Docker image with a pre-configured GIVE server and GIVE web components, to quickly set up and run the genome browser without installing any other dependencies.

The seamless provision of the features described above was not an easy task. Although users do not need to fully understand the underlying technology, the GIVE team has developed novel algorithms and data structures for data management, communication and memory management to make the genome browser interactive and responsive. These include two new tree-based data structures named Oak and Pine, which are wrapped inside the GIVE library, to make data transfer faster and memory efficient (Fig. 1e, f). Oak is designed to handle genome annotation data that is in BED format and sparse, and Pine handles dense data in BigWig format. The team also developed a ‘withering’ algorithm to manage the memory efficiently. These algorithms make the GIVE browser more robust by only transferring the required data at its requested resolution and by re-using the data previously transferred to the web browser.

With this type of technology working ‘behind the scenes’, GIVE is able to provide three options for the user to set up a customized genome browser in only a few minutes (Fig. 1), as illustrated in GIVE’s online demonstration. The first and easiest option is to search for the data of interest in the GIVE Data Hub and to export this data to HTML using the HUG interface, and then to view it or share it with the public by inserting the code generated by HUG into a website. In the second scenario, if the data is not available in the data hub, the user can send a request to the GIVE team to include the data and then visualize it once the metadata is available on the GIVE Data Hub. The third option is to set up a local version of the GIVE server using the GIVE-Docker or GIVE-Toolbox, and thus the data on the local server can be visualized on the genome browser in the same way as for public data.

## Outlook and future perspectives

Every day, thousands of laboratories and institutions are generating and collecting huge amounts of genomics and other omics data. Being able to interactively show, compare and integrate data from multiple sources is crucial for making the data valuable. Besides the commonly used genomic portals built by major genomic centres and consortia, many laboratories are in great need of their own websites in order to release their data and analysis results. At this critical juncture, GIVE provides the scientific community with a very timely modular, versatile and efficient library for creating an ultralight, embeddable and fully functional genome browser that can be embedded on personal websites. GIVE encapsulates novel data communication and visualization components, including new data structures and memory management algorithms that facilitate efficient data transfer between data servers and browsers. With this powerful platform, we anticipate that more and more scientists and even amateurs will be publishing and sharing their GIVE codes, and a community-driven GIVE plug-in store, similar to the example of Cytoscape Apps, will eventually revolutionize the application of the genome browser and the way in which people view, share and analyse genomics and other omics data.

## Abbreviations

ChIP-seq: Chromatin immunoprecipitation sequencing
GIVE: Genomic interaction visualization engine
HUG: HTML universal generator
